# Multi-Epitope DNA-Based Feline Immunodeficiency Virus Vaccine Construct Designed by Immunoinformatic and Machine Learning Tools as a Surrogate Model for HIV Vaccine Development

**DOI:** 10.3390/pathogens15030341

**Published:** 2026-03-23

**Authors:** Tyler Michalka, Abid Ullah Shah, Tiffany Liang, Maged Gomaa Hemida

**Affiliations:** Department of Veterinary Biomedical Sciences, Lewyt College of Veterinary Medicine, Long Island University, Brookville, NY 11548, USA; tyler.michalka@my.liu.edu (T.M.); abidullah.shah@liu.edu (A.U.S.); tiffanyl8281@gmail.com (T.L.)

**Keywords:** feline immunodeficiency virus (FIV), human immunodeficiency virus (HIV), multi-epitope DNA vaccine, artificial intelligence, immunoinformatics

## Abstract

Feline immunodeficiency virus (FIV) is a lentivirus that exhibits significant structural and pathological similarities to human immunodeficiency virus (HIV), establishing it as a valuable model for HIV vaccine development. In this study, artificial intelligence (AI) and immunoinformatics were employed to design a novel multi-epitope DNA vaccine targeting conserved regions of the FIV *gag*, *pol*, and *env* genes. Predicted B-cell and T-cell epitopes were evaluated for their capacity to induce strong immune responses while minimizing allergenic or toxic effects and were linked to the immune adjuvant PADRE. Structural analysis indicated that the vaccine construct is stable, soluble, and biocompatible, with a well-folded tertiary structure that binds Toll-like receptor 9 (TLR9) and elicits robust humoral and cellular immune responses. These findings identify a promising FIV vaccine candidate and provide insights for the development of next-generation HIV vaccines.

## 1. Introduction

Human immunodeficiency virus (HIV) remains a major challenge in infectious disease management largely due to its high mutation rates that help it resist current antiretroviral therapy. Resistance-associated mutations often arise in a single host but can subsequently spread between individuals, enabling the transfer of therapy-resistant strains and complicating effective treatment and vaccine development [1,2,3,4]. From a global perspective, this shows a growing health crisis in which antiretroviral therapy resistance is estimated to be the result of up to 10 million deaths annually by 2050 [5].

The growing resistance to current antiretroviral therapies highlights the need for alternative approaches to treatment, including next-generation vaccines such as multi-epitope vaccines [6]. In HIV research, feline immunodeficiency virus (FIV) is a valuable comparison model. FIV, a lentivirus, produces HIV-like symptoms in felines, including progressive immune system deterioration through a decrease in CD4+ T cells [7]. Both viruses have conserved lentiviral gene products: *gag*, *pol*, and *env*, which underpin a shared genomic organization and give rise to similar structural and functional features [7,8]. Although FIV causes less severe disease, FIV’s genetic structure, disease progression, and elicited immune response make it a highly effective model for studying HIV treatment strategies [9,10].

Ongoing research on FIV has identified several antiviral mechanisms that make it a useful model for studying lentiviral immunity and drug resistance [8,11,12]. In addition to this, FIV was the first lentivirus in which antiviral resistance was observed, providing significant insight into the design of HIV therapy [7]. However, effective FIV vaccines remain limited, and the immune mechanisms that protect against mutations are not yet fully understood [12]. With recent advancements in artificial intelligence (AI), there is potential to use AI-based tools to efficiently develop a targeted vaccine against FIV. This can be done by predicting the optimal epitopes, designing protein structures, and predicting the immune system’s response [6,10,13,14,15].

Traditional vaccine development using attenuated or inactivated viruses has been successful for many pathogens but has proven challenging for rapidly mutating viruses [16]. Lentiviruses, such as FIV and HIV, show these challenges due to high mutation rates and integration into the host genome, which limit the effectiveness of these vaccines [8]. Advances in artificial intelligence are promising for addressing limitations in traditional vaccine development, such as time and cost, and enable rapid screening of datasets to identify epitopes, model structure, and ligand-receptor interactions, as well as predict a host’s immune response to the vaccine [17,18,19,20]. These technological advancements have the potential to drive breakthroughs in treatment, especially when paired with analogous models like FIV.

Monitoring of the viral genomes through genome sequencing mining of viral diseases of livestock/companion animals and birds on the nucleotide and amino acid levels provides an important privilege in the design of next-generation vaccines and antiviral therapy [21,22,23,24,25,26,27,28,29,30,31]. Integration of genome sequencing, machine learning and generative artificial intelligence tools provides great opportunities to design next-generation vaccines and antiviral drugs as well as study the functions of some key viral proteins in the molecular pathogenesis of viral diseases affecting humans, animals and birds [21,32,33,34]. This approach increases the precision and accuracy in the vaccine/drug design and repurposing, and reduces the time required for the approval of newly designed vaccines [21,32,33,34].

**Figure 1:**
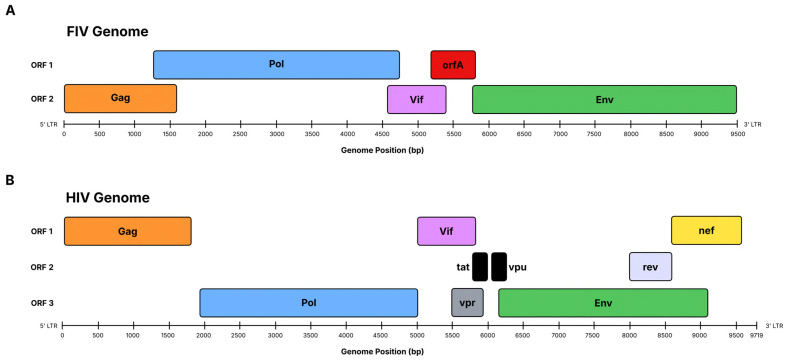
Comparative genomic organization of; (**A**) feline immunodeficiency virus (FIV) and (**B**) human immunodeficiency virus (HIV). Both viruses share conserved *gag*, *pol*, and *env* genes, as well as genes that regulate replication and immune evasion. The structural and functional similarities highlight why FIV serves as a surrogate model for HIV vaccine design. This schematic was created using Lucidspark and previous organizational models [11,35,36,37].

The present study aimed to identify highly conserved regions (*gag*, *pol*, and *env*) across known FIV genome isolates that have functions analogous to those of HIV in viral entry, assembly, structure, and replication. A comparison of viral genomic organization, highlighting the conserved structural genes among retroviruses, is shown in Figure 1. The multi-epitope DNA vaccine construct demonstrates high antigenicity, structural stability, strong affinity for the body’s innate immune receptors (TLR9), and a robust immune response. These results support this vaccine’s potential to serve not only as an FIV vaccine candidate but also as a computational model to inform HIV vaccine development.

## 2. Materials and Methods

### 2.1. Retrieval of the FIV Sequences and the Multiple Sequence Alignment (MSA)

Full amino acid sequences of 112 FIV isolates (see Table S1) were obtained from the NCBI GenBank database. Only sequences containing complete coding regions for the *gag*, *pol*, and *env* genes were included in the analysis. Partial sequences, sequences containing ambiguous residues, and duplicate isolates were excluded to ensure consistency during downstream analyses. Multiple sequence alignment (MSA) was performed using Geneious v2023.1 and Clustal Omega V.1.2.4 with default parameters, including a gap-opening penalty of 10 and a gap-extension penalty of 0.2. The aligned regions of the genes and derived consensus sequences for *pol*, *gag*, and *env* were used to compare the FIV Petaluma strain [M25381.1] epitopes with the consensus sequence and with those of the 111 other isolates.

### 2.2. Prediction and Mapping of Some Common T Cell Epitopes Across Some Key Target FIV Proteins

Major histocompatibility complex I (MHC I) epitopes were predicted by using amino acid sequences for each gene of the FIV Petaluma strain in the IEDB MHC I-Binding Prediction Tool to obtain 9-mers. Characterization of feline MHC I is poor, resulting in the utilization of DLA-8803401. The use of this allele was justified by comparing expressed FLA molecules [38] with previously used DLA molecules [39]. To evaluate the suitability of this substitution, pairwise sequence comparisons were performed between expressed feline leukocyte antigen (FLA) class I alleles and canine DLA-88 alleles. The Blastp analysis demonstrated approximately 72–77% sequence identity across the aligned extracellular domains (Table S3). The observed identity values reflect differences in overall sequence length and non-homologous regions between species; however, the α1 and α2 domains that form the peptide-binding groove remain highly conserved, supporting the use of dog leukocyte antigen (DLA-88*03401) [Acc. NM_001014767.1] as a surrogate allele for preliminary MHC-I epitope prediction. Resulting epitopes were ranked according to the percentile binding score generated by the IEDB prediction algorithm, and peptides with a percentile rank ≤ 2.0 were retained for further analysis.

Each gene amino acid sequence of the FIV Petaluma strain was inserted into IEDB MHC II Binding Predictions to obtain 15-mers. The use of human leukocyte antigen—DR isotype (HLA-DR) [DRB1*01:01, DRB1*04:01, DRB1*07:01, DRB1*11:01, DRB1*15:01]—was justified by utilizing NCBI Blastp to compare the feline major histocompatibility complex II (MHC II) DRB β1 extracellular domain (FLA-DRB) [Acc. U51527] to homo sapiens reference proteins. The analysis demonstrated approximately 75–78% sequence identity within the peptide-binding β1 domain, with highly significant E-values (<10^−32^) (Table S4). Because the β1 domain forms the primary peptide-binding groove responsible for antigen presentation, this level of conservation supports the use of HLA-DRB1 alleles for preliminary MHC II epitope prediction. Similar surrogate-allele approaches have been widely used in immunoinformatics-based vaccine design when species-specific MHC datasets are unavailable [40]. Predicted epitopes were ranked according to the percentile binding score generated by the IEDB prediction algorithm, and peptides with a percentile rank ≤2.0 were retained for further analysis.

### 2.3. Prediction and Mapping of Some Common B-Cell Epitopes of the Target Viral Proteins

Each gene of the FIV Petaluma strain was inserted into ABCpred to identify linear B-cell epitopes using the default 16 amino acid (aa) parameters. Results ≥ 0.80 were retained and cross-validated using BepiPred 2.0, which uses amino acid propensity scales to suggest antibody-accessible epitopes on a high-to-low scale. Epitopes receiving a high-moderate score from Bepipred were preferred in selection. Surface exposure was confirmed using ColabFold to generate the 3D structures of *gag*, *pol*, and *env*. Each of these genes was inserted into UCSF ChimeraX, and epitopes exhibiting surface exposure were identified as those with a solvent-accessible surface area exceeding 300 Å^2^, indicating sufficient accessibility for immune recognition.

### 2.4. The Immunological and Safety Profiling of the Mapped FIV Epitopes Across the Key Proteins

T- and B-cell epitopes were assessed for antigenicity, allergenicity, and toxicity using VaxiJen v2.0, AllerTOP v2.1, and ToxinPred, respectively. Epitopes were selected based on high antigenicity and a lack of allergenicity and toxicity. The criteria were only epitopes with high antigenicity scores (≥0.4), non-allergenic epitopes, and non-toxic epitopes.

### 2.5. Prediction of the Ability of Mapped T-Cell Epitopes for the Feline Cytokine Production and Cross-Reactivity

The MHC II T-cell epitopes were analyzed using IL4Pred to predict interleukin-4 (IL-4) production. The hybrid prediction method and an SVM threshold of 0.2 were used on this server, with positive values indicating successful induction. The potential of selected T-cell epitopes to induce interferon gamma (IFN-γ) was evaluated using the IFNepitope server. Hybrid prediction approach and a support vector machine (SVM) threshold of 0.2 were used to identify IFN-γ induction potential. Epitope cross-reactivity with non-redundant protein sequences of Felis catus (taxid:9685) was evaluated using NCBI BLASTP to assess homology with host proteins.

### 2.6. In Silico Construction of the FIV Multiepitope DNA Vaccine

All B and T cell epitopes were connected using linkers, along with adjuvants Feline β-defensin 1 and PADRE, to form a multi-epitope construct. Each epitope and adjuvant was joined using linkers such as KK, HE, GSGSG, and GPGPG. Each linker was added to facilitate proper epitope separation and folding, while minimizing structural interference between epitopes. At the C-terminal end, a histidine tag was incorporated into the construct.

### 2.7. Analysis of the Physicochemical and the Peptide Solubility Properties of the FIV Vaccine Construct

By submitting the FASTA sequence, the transmembrane regions of the protein were predicted using TMHMM-2.0’s standard TMHMM prediction. NETPHOS-3.1 was used to identify the phosphorylation sites, and the protein’s hydrophilicity was evaluated using Expasy. Glycosylation sites of the protein were determined using NetNGlyc 1.0. Default parameters were used for each program.

### 2.8. Prediction of the Secondary and Tertiary Structures of the Potentially Expressed Proteins

The secondary structure prediction of the peptide was performed using NPS, while the protein’s tertiary structure was generated using ColabFold and visualized in ChimeraX. The pre-optimized protein was analyzed with ProSA, and further structural analysis was performed with MolProbity. The protein structure was optimized using GalaxyRefine and rerun through both ProSA and MolProbity [41].

### 2.9. Molecular Docking and Simulation

The Toll-like receptor 9 (TLR9) was selected to engage the innate immune system and stimulate antigen presentation (Gupta). The full-length feline TLR9 molecule was obtained from the NCBI database (Table S3) and converted to a peptide in Expasy, followed by tertiary structure prediction in ColabFold. The docking between the multi-epitope protein TLR9 was simulated using HDock by uploading the PDB file of the sequence with no specific restraints and global docking, with the chemical interactions being interpreted by PDBePISA.

### 2.10. In Silico Prediction of the Immune Stimulation Simulation of the Designed FIV Vaccine Construct

The peptide’s immune response was simulated using C-Immsim. Initial vaccination was administered on day 7 (step 21), followed by boosters at day 21 (step 63) and day 35 (step 105). The immune response was observed through each administration until day 70 (total simulation steps = 1000) [40].

### 2.11. In Silico Cloning of the Multiepitope FIV Vaccine Construct

The plasmid pcDNA3.1+ was obtained from Addgene, and restriction enzymes EcoRI and XhoI were used to insert the peptide using Snapgene.

### 2.12. Software

All bioinformatic tools and web servers used in this study, including version numbers and access links, are summarized in Table 1.

## 3. Results

### 3.1. FIV Multiple Sequence Analysis

Viral sequences were collected from the NCBI GenBank. Using Geneious, multiple sequence alignment was performed, highlighting conserved regions of the sequence that could be targeted across multiple isolates. The aligned viral sequences were used to create a consensus sequence for further evaluation and comparison to predicted epitopes of the Petaluma strain [M25381.1]. All valid predicted B and T-cell epitopes are shown in Table S2.

### 3.2. Mapping Some B-Cell Epitopes Across the Key Proteins of the FIV Genomes

The *gag*, *pol*, and *env* genes of the FIV Petaluma strain were used to predict linear B-cell epitopes using ABCpred, followed by BepiPred for further verification of antibody accessibility. Protein conformational analysis using ColabFold and UCSF Chimera predicted surface-exposed epitopes, with epitopes exhibiting surface exposure greater than 300 Å^2^ considered accessible for immune recognition. The epitopes were tested for antigenicity using VaxiJen, with a threshold score ≥0.4 indicating a probable antigen. Allergenicity was predicted using AllerTOP v2.1 and toxicity using ToxinPred. Epitopes were screened against Felis catus proteome (taxid:9685) using NCBI BLAST v.2.17, and epitopes with significant homology to the host protein were excluded. From the initial set of predicted epitopes, candidates that met all predefined selection criteria (MHC binding percentile ≤2.0, antigenicity ≥0.4, non-allergenic, non-toxic, and non-homologous to host proteins) were retained. These filtered epitopes were then ranked based on antigenicity score and immunological relevance, and the top six epitopes from each category were selected for inclusion in the vaccine construct (Table 2). The predicted antigenicity, allergenicity, and toxicity are shown in Table S2.

### 3.3. Mapping and Selection of Some Cytotoxic T Lymphocyte (CD8^+^) Epitopes Across the Key Proteins of the FIV Genomes

The *gag*, *pol*, and *env* genes of the FIV Petaluma strain were used to predict cytotoxic T lymphocyte (CTL) via the IEDB MHC I-Binding Prediction server. The canine allele was selected based on conserved sequence similarity to feline leukocyte antigen (FLA) molecules and its previous use in an immunoinformatic study [39]. Alignment of expressed feline leukocyte antigen (FLA) class I molecules with canine DLA-88 alleles demonstrated sequence identity values ranging from 72–77% across the aligned extracellular domains, with non-homologous regions largely present outside the α1 and α2 binding domains, which are crucial for epitope prediction. Epitopes were ranked, with peptides receiving a percentile rank ≤2.0 retained, and redundant core peptides removed. The same criteria used for B-cell epitope prediction were applied to predict antigenicity, allergenicity, toxicity, and cross-reactivity of the CTL epitopes. Chosen epitopes are shown in Table 3, while all valid epitopes are shown in Table S2.

### 3.4. Mapping and Selection of Some Helper T Lymphocyte (CD4^+^) Epitopes Across the Key Proteins of the FIV Genomes

The genes from the FIV Petaluma strain were entered into the IEDB MHC II Binding Prediction server to identify epitopes with a percentile rank ≤ 2.0. MHC II alleles HLA-DR [DRB1*01:01, DRB1*04:01, DRB1*07:01, DRB1*11:01, DRB1*15:01] were justified by utilizing NCBI Blastp to compare the feline MHC class II DRB β1 extracellular domain (FLA-DRB). The extracellular β1 domain of feline FLA-DRB demonstrated approximately 75–78% sequence identity with human HLA-DRB1 alleles, with highly significant BLASTp E-values (<10^−32^) (Table S4). Using these predicted epitope sequences, each was then tested for allergenicity, antigenicity, and toxicity in the same manner as CD8^+^ and B-cell epitopes. The cytokine induction potential of the MHC II T-cell epitopes was analyzed using IL4Pred and IFNEpitope. All of the selected epitopes demonstrated production of IL4, which suggests the construct’s ability to induce a Th2 response [65]. Although only two epitopes were predicted to induce IFN-γ, later immune simulation demonstrated a robust potential to produce IFN-γ. Epitopes used in the construct are shown in Table 4, and full epitope results are available in Table S2.

### 3.5. Selection of the Top-Ranked Epitopes and the Design of the FIV-DNA Vaccine Construct

**Figure 2:**
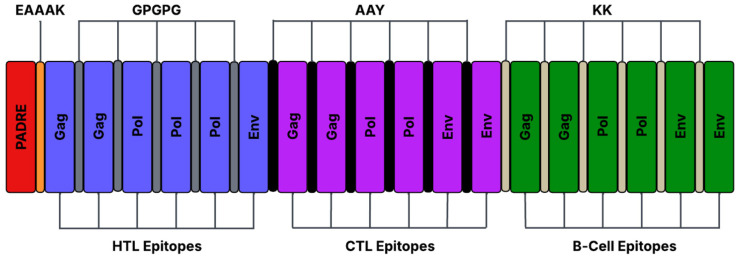
Schematic representation of a multi-epitope vaccine construct targeting FIV. The construct is arranged from C-terminus (left) to N-terminus (right). It contains the immunostimulatory adjuvant PADRE, along with sequential B- and T-cell epitopes from the conserved regions of the *pol*, *gag*, and *env* genes of FIV. The epitopes are linked using rigid linkers (EAAAK) and flexible linkers (GPGPG, AAY, and KK) to allow for proper tertiary folding of the construct. A histidine tag was added to the N-terminus of the construct to facilitate protein purification.

From the filtered epitope pool, the top six epitopes in each category were selected based on their combined immunological properties, including antigenicity score, MHC-binding percentile rank, cytokine-induction potential, and lack of allergenicity or toxicity. This number was selected to balance epitope diversity and construct size, while ensuring representation across the three major structural genes (*gag*, *pol*, and *env*). Due to limited valid epitopes, only one HTL env epitope was used, with replacement by the next highest-ranked pol epitope. The final vaccine construct included 18 epitopes: six B-cell epitopes, six cytotoxic T-lymphocyte (CTL) epitopes, and six helper T-lymphocyte (HTL) epitopes. Highly immunogenic epitopes were linked using EAAAK, GPGPG, AAY, and KK. PADRE was added to the N-terminus of the peptide as an adjuvant to stimulate a helper T-cell response and increase vaccine potency [66]. Although use of PADRE as an adjuvant is better described in relation to human HLA-DR molecules, its ability to induce a CD4^+^ T-cell response in vaccines has been demonstrated across species due to the conserved nature of the MHC II binding groove [66,67]. Due to the feline FLA-DR molecules’ structural homology with other mammalian MHC-II proteins, PADRE was included as a broadly reactive helper epitope to improve predicted immunogenicity. A histidine tag was then added at the C-terminus to facilitate protein purification and detection. The full construct is shown in Figure 2.

### 3.6. The Physicochemical Properties of the Designed FIV-DNA Vaccine Construct

**Figure 3:**
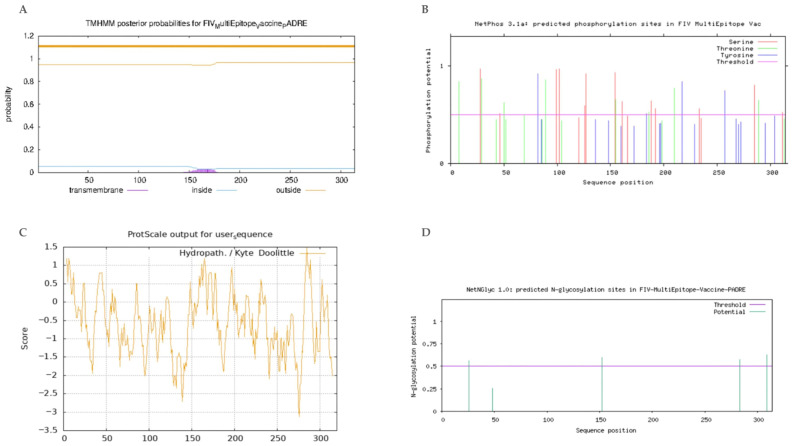
Results of physicochemical property analysis of vaccine protein. (**A**) Transmembrane prediction results; (**B**) Predicted phosphorylation sites; (**C**) Hydrophilicity prediction; (**D**) Predicted glycosylation sites.

The physicochemical and solubility properties of the vaccine construct were assessed to evaluate suitability for stability, expression, and immunogenic performance. Expasy Protparam results indicated that the construct has a molecular weight of ~35.4 kDa (320 amino acids) and a basic pI of 9.91, indicating interaction with negatively charged immune cell membranes. Protparam also indicated a grand average hydropathicity (GRAVY) score of −0.6 and a stable protein value of 29.71. Full analysis in Table S4. DeepTMHMM2.0 predicted no transmembrane regions in the vaccine protein, with residues classified as extracellular. This supports the construct’s non-membrane-bound status and demonstrates potential accessibility for antigen processing [68] (Figure 3A).

NetPhos-3.1 identified 48 phosphorylation sites distributed across residues of the construct (Figure 3B). The numerous predicted phosphorylation sites suggest that the vaccine protein may have increased immunogenicity via activation of antigen presentation by innate immune cells, enhanced T-cell activation, and B-cell proliferation [69]. The analysis by Expasy revealed that the vaccine protein is primarily hydrophilic and soluble in water, with a hydrophilic peak of −3.1 and a hydrophobic peak of 1.5 (Figure 3C). The predicted high solubility indicates antigen dissolution and presentation to the immune system [42]. NetNGlyc 1.0 predicted four glycosylation sites that marginally exceed the threshold of 0.5, indicating the protein may not have any glycan-mediated epitope masking. (Figure 3D) [70].

### 3.7. Results of the Predicted Antigenicity and Allergenicity of the FIV-DNA Vaccine Construct

Using VaxiJen 2.0, the antigenicity of the vaccine peptide with adjuvants was estimated to be 0.7192. When the software was used solely with peptides (without adjuvants), the antigenicity was estimated at 0.7288. This shows that the vaccine, with or without adjuvants, is antigenic. AllerTOP 2.1 was used on the vaccine with and without adjuvants and was confirmed to be non-allergenic. The vaccine was confirmed to be non-toxic, using ToxinPred, in its complete and adjuvant-free forms as well.

### 3.8. The Secondary Structure of the Multiepitope FIV-DNA Vaccine Construct

**Figure 4:**
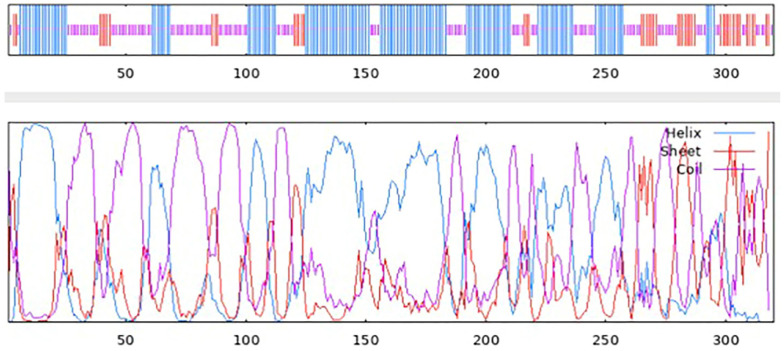
Secondary structure analysis of protein, where alpha helix is represented by helix, extended strands are represented by sheet, and random coils are represented by coil.

The NPS online software v.3 was used to examine the protein’s secondary structure, indicating the composition to be 45.31% alpha helix, 15% extended strands, and 39.69% random coils (Figure 3). This suggests the construct is partially ordered due to the alpha helix content, but conformationally adaptable based on the coils and strands [71]. Secondary structure analysis by NPS is shown in Figure 4.

### 3.9. The Tertiary Structure of the Multiepitope FIV-DNA Vaccine Construct and Feline TLR9

**Figure 5:**
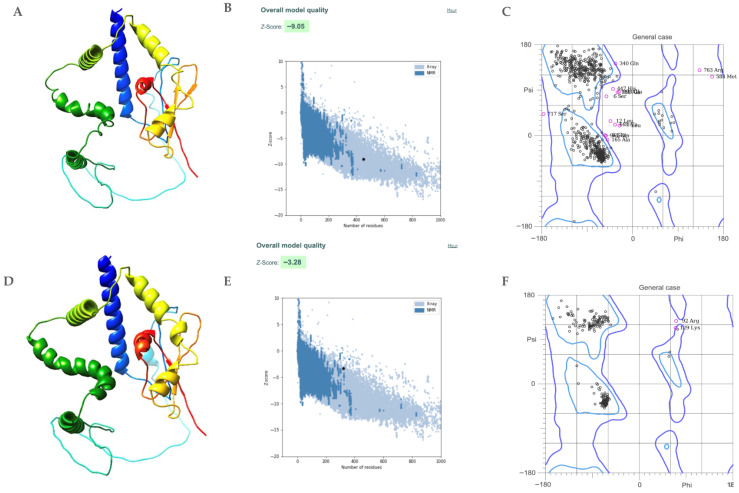
Displays the tertiary structure and analysis of the protein structure. (**A**) Pre-optimized protein structure; (**B**) Pre-optimized Z-score residue analysis; (**C**) Pre-optimized Ramachandran plot; (**D**) Post-optimization protein structure; (**E**) Post-optimization Z-score residue analysis; (**F**) Post-optimization Ramachandran plot.

The predicted tertiary structure of the protein was generated using ColabFold, and the highest-scoring model was selected for further visualization in ChimeraX (Figure 5A). The pre-optimized protein received a Z-score of −9.05 using ProSA (Figure 5B), and further analysis of the structure was performed in MolProbity, which analyzed the Ramachandran plots (Figure 5C) and showed that 66.04% of the protein had favorable geometry. The protein structure was optimized using GalaxyRefine and rerun through both ProSA and MolProbity. The resulting Z-score was −3.28, with a significantly improved 97.48% favored protein geometry and two Ramachandran outliers. The refinement substantially improved the geometry and quality of the construct, enabling further analysis. The predicted feline TLR9 structure was obtained from Colabfold and demonstrated high overall confidence, with a mean predicted local distance difference test (pLDDT) score of approximately 89 across the sequence. Predicted aligned error (PAE) analysis showed low intra-domain error values (~5–8 Å) with higher uncertainty observed between domains (~20–30 Å). Protein tertiary structure and analysis are shown in Figure 5.

### 3.10. Results of the Molecular Docking with the Ligand-Binding Domain of the Feline TLR9

**Figure 6:**
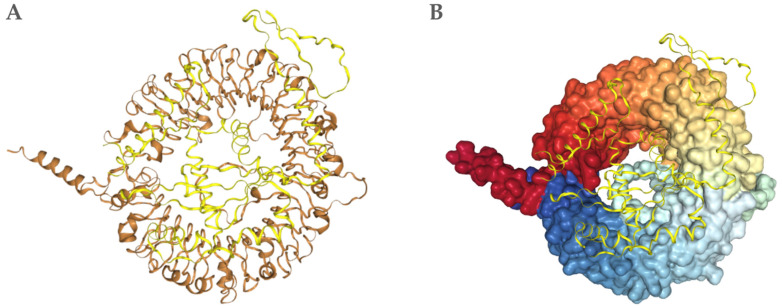
Molecular docking visualization of the vaccine with feline TLR9. (**A**) The receptor (brown) is shown in ribbon configuration, while the vaccine construct (yellow) is shown in ribbon configuration, occupying the TLR9 receptor-binding domain. (**B**) Surface representation of TLR9 colored by structural domains, highlighting the bonding interface with the ligand (yellow).

The refined tertiary structure of the vaccine construct was validated using ProSA before docking analysis. The vaccine construct and the binding domain of feline toll-like receptor (TLR9) were then submitted to HDock for docking, followed by PDBePISA for post-docking analysis. Toll-like receptor 9 (TLR9) was selected because it plays an important role in innate immune activation following recognition of unmethylated cytosine-phosphate-guanine (CpG) motifs commonly present in plasmid DNA used in DNA vaccines. Therefore, in the context of this study, TLR9 activation is primarily expected to occur through recognition of CpG motifs within the vaccine plasmid rather than direct interaction with the expressed protein product. As a result, the docking results constitute a structural assessment and should not be interpreted as evidence that the expressed protein functions as a direct TLR9 agonist. HDock predicted 10 complexes; model 1 (Figure 6) had the lowest docking energy (−304.77) and the highest confidence score (0.9567), indicating a favorable binding orientation. PDBePISA predicted a total interface area of 1933 Å^2^ and a ΔG of −8.7 kcal/mol upon complex formation, which suggests an energy-favorable interaction between the construct and TLR9. Structural analysis revealed 9 hydrogen bonds and 3 salt bridges between the ligand and TLR9, indicating a strong noncovalent interaction between the pair. The following are the binding residues between the construct and TLR9: Hydrogen bonds: A:GLN367—B:TYR172, A:ARG601—B:VAL211, A:ARG656—B:ALA214, A:ARG726—B:TYR217,A:GLN38—B:LYS10, A:ASN309—B:ARG142, A:SER365—B:ARG149, A:MET311—B:ARG179. Salt bridges: A:ARG338—B:GLU180. See Figures S1 and S2 for additional TLR9 docking and analysis information.

### 3.11. The Immune Stimulation Results of the Designed Multiepitope FIV-DNA Vaccine Construct

**Figure 7:**
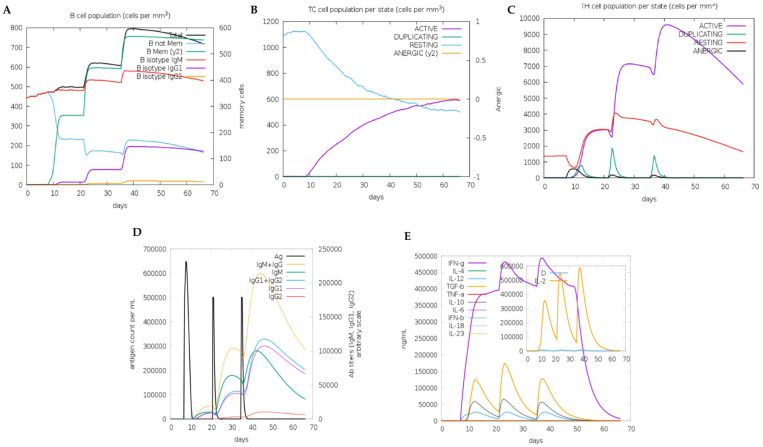
Immune stimulation results of the vaccine construct using C-Immsim. (**A**) B-cell proliferation following repeated doses. (**B**) Antibody titers (IgM, IgG) following exposure to antigen. (**C**) Helper and memory T-cell production. (**D**) Cytotoxic T-cell activation. (**E**) Cytokine profile showing mainly increased IFN-γ and IL-2.

The C-ImmSim server was used to simulate how the immune system would respond after three administrations of the vaccine, 14 days apart, over the course of 70 days. Because C-ImmSim is a rule-based computational model, the results represent predicted immune response trends rather than experimental measurements. The B-cell population (Figure 7A) showed a rapid increase in total B-cell and memory B-cell populations following the initial administration on day 7, with subsequent boosters on days 21 and 35. The sustained memory B-cell population (Figure 7A) and class switching between immunoglobulin M (IgM) and immunoglobulin G (IgG) suggest an effective immune response and enhanced antigen presentation. Production of antibodies, mainly IgM and IgG (Figure 7B), showed the potential to elicit long-lasting immunity to the vaccine construct. Substantial activation of T-helper cells (Figure 7C), as well as moderate activation of cytotoxic T-cells (Figure 7D), suggests a robust CD4^+^ immune response with a gradual increase in CD8^+^ cells over time. The activation of the cytokines IL-2, IL-4, and IL-10 indicates a moderate Th2-mediated immune response (Figure 7E). The immune simulation predicted elevated IFN-γ levels following booster administrations, suggesting a potential T-helper cell 1 (Th1)-associated immune response. The computer-generated predicted activation of both cellular and humoral immunity should be interpreted as hypothetical trends requiring experimental validation. These predicted immune trends are consistent with previous studies showing that effective control of FIV infection is associated with virus-specific T-cell responses and IFN-γ production [12,72,73].

### 3.12. In Silico Cloning Results of the Final Recombinant Vaccine Construct

**Figure 8:**
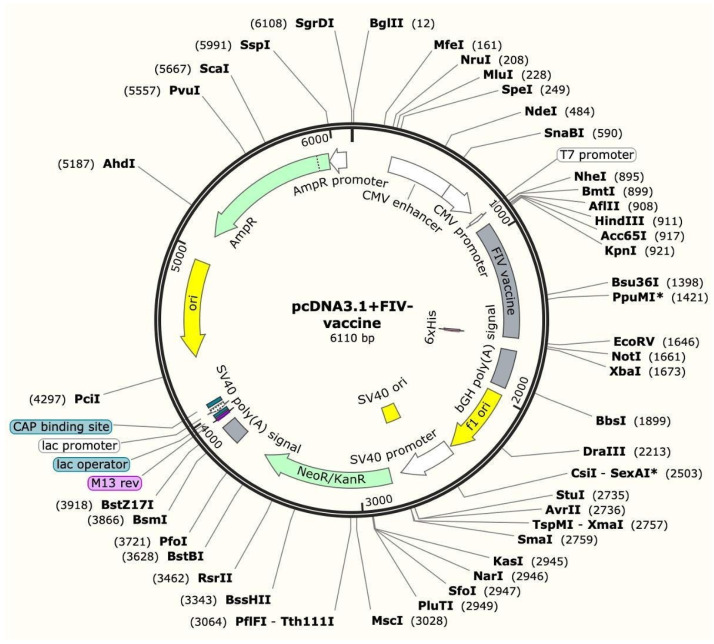
In silico cloning of the multi-epitope vaccine construct into the pcDNA3.1(+) mammalian expression vector. The construct was introduced between the EcoRI and XhoI restriction sites.

The final multi-epitope vaccine construct was cloned in silico into the mammalian expression vector pcDNA3.1(+) vector using EcoRI and Xhol restriction sites. The recombinant plasmid was designed and verified in SnapGene to ensure proper insertion into the expression vector. Cloning results are shown in Figure 8.

## 4. Discussion

This study aimed to use artificial intelligence-assisted immunoinformatics to develop a multi-epitope DNA vaccine targeting conserved regions of feline immunodeficiency virus (FIV) *gag*, *pol*, and *env* genes. Using 112 FIV isolates, the vaccine construct effectively targeted genes from known circulating isolates to maximize coverage. The developed vaccine construct exhibited physicochemical and structural stability, high antigenicity, and a tertiary structure suitable for downstream analysis. The construct’s capability to elicit robust humoral and cellular immune responses in silico was demonstrated through immune simulation using a prime-booster vaccination strategy, yielding strong antibody production with class switching, sustained helper T-cell responses, a significant cytotoxic T-cell response, and a cytokine profile indicative of effective immune activation.

The immune stimulation findings suggest that the vaccine preferentially promotes cell-mediated immunity, with a strong CD8^+^ T-cell response associated with robust IFN-γ responses to each booster. This cellular immune profile is particularly important for combating retroviral infections, such as FIV and HIV, which can integrate rapidly into host genomes. As such, neutralizing antibodies will reduce viral entry and the establishment of infection through early recognition of the virus, but a cytotoxic response is necessary for the clearance of rapidly infected cells and is crucial for effective immune control of retroviruses [4,12,72]. FIV and HIV vaccine literature emphasizes that durable viral control correlates with the magnitude of the virus-specific CD8^+^ T-cell response, with IFN-γ playing a central role in promoting the cytotoxic response [4,12,73]. Further vaccine research on FIV demonstrates that anti-FIV T-cell immunity is crucial for protection against FIV, and possibly HIV-1 [72].

The cytokine profile, predominantly IL-2, IL-4, and IL-10, demonstrates the induction of an effective Th2-mediated humoral immunity necessary for antibody production, maturation, and immune memory, as identified in previous multiepitope vaccine strategies [65,74]. The production of persistent memory B cells, with antibody production and IgM-to-IgG class switching, is a hallmark of successful vaccination strategies [65,74,75]. The inclusion of PADRE likely further supported Th2-cell activation and antigen presentation, consistent with its role in previously described multiepitope vaccines [67,76]; however, its immunostimulatory effect in felines remains a prediction awaiting experimental validation. These predicted immune trends are consistent with previous studies demonstrating that effective control of FIV infection is associated with virus-specific T-cell responses and IFN-γ production, which play an important role in antiviral immunity [72,73] and support the vaccine’s potential to induce an effective immune response.

The design strategy used in this study closely follows the framework of previous immunoinformatic vaccines targeting viral pathogens, including feline infectious peritonitis, infectious bronchitis virus, human papillomavirus, and cyprinid herpesvirus. As in these studies, the inclusion of epitopes from multiple proteins broadens immune coverage and reduces the likelihood of escape [39,41,77,78]. Lentiviral and retroviral vaccine designs are often unable to maintain effectiveness due to the high level of antigenic diversity and ongoing mutations. To combat this, 18 epitopes were selected targeting the more conserved regions of the *gag* and *pol* genes, enabling a durable immune response that is less dependent on viral strain. In contrast, env-derived (envelope) epitopes can elicit antibody production despite antigenic diversity. The balance of conserved and variable gene targets has also been described in studies of feline immunodeficiency and immunodeficiency-like viruses [77,79].

Immunological and safety screening is another advantage of immunoinformatic-driven vaccine design. Filtering all epitopes based on antigenicity, allergenicity, toxicity, and cross-reactivity enables early removal of potentially harmful proteins. Selection based on these requisites has been identified as a critical step in improving translational feasibility and in reducing adverse immune outcomes [44].

Structural and physicochemical analysis further supports the feasibility of this vaccine construct. The immunoinformatics analysis indicated that the construct is stable, soluble, and suitable for immune recognition, while maintaining a well-folded, refined tertiary structure. Proper folding is critical for epitope recognition and processing by the innate immune system, a step that is critical for downstream activation of adaptive immunity and successful vaccination [39,41]. The vaccine’s stable and accessible structure provides a reliable foundation for evaluating its interaction with a receptor, TLR9.

The design vaccine construct demonstrated a stable, energetically favorable interaction with toll-like receptor 9 (TLR9), as evidenced by hydrogen bonds and salt bridges. Stimulation of this receptor has been shown to play a role in innate antiviral responses in felines [80,81], therefore supporting the previously mentioned downstream activation of adaptive immunity. As TLR9 recognizes unmethylated CpG motifs in DNA within endosomes, activation in DNA vaccines is expected to result primarily from CpG sequences in the plasmid backbone rather than from the expressed protein, rather than through direct interaction with the expressed protein product. These results demonstrate a mechanistic basis for this vaccine inducing an immune response in felines and do not provide definitive evidence that the construct functions as a direct TLR9 agonist.

Several limitations of this study should be acknowledged, many of which are common among immunoinformatic-based multi-epitope vaccine design. Expression of this vaccine in vivo cannot be fully accounted for by computational predictions, as post-translational modifications, host immune system variability, biological complexities, and many other factors contribute to its expression. Epitope selection, structural behavior, receptor interactions, and immune responses are thus largely host-dependent, and the computational findings in this study serve only as preliminary evidence for experimental validation. The construct’s predicted structure represents a static protein conformation, and in silico design does not fully capture dynamic structural changes, glycosylation, or intracellular processing that may occur in vivo. This limitation carries over into ligand-receptor modeling, where both structures are static and unable to capture the complex binding and conformational changes present in either molecule upon interaction. Immune simulation results are rule-based approximations of immune responses and can only represent theoretical trends based on a defined model. These limitations highlight the need for experimental validation and should be considered when interpreting the potential translational application of this vaccine construct.

Another limitation relates to the use of surrogate MHC alleles for epitope prediction. Due to the limited availability of feline-specific prediction models, canine DLA-88 and human HLA-DRB alleles were used to approximate feline MHC binding preferences. Although sequence comparisons demonstrated substantial conservation within the peptide-binding domains of these molecules, cross-species substitutions may introduce bias, as allele-specific peptide-binding motifs can differ between species. As a result, some predicted epitopes may display different binding affinities when presented by native feline MHC molecules. Similar constraints have been noted in multiepitope vaccine studies [39,41,78].

Several practical challenges should also be considered when translating a multi-epitope DNA vaccine from in silico design to real-world application. While in silico analyses are valuable for identifying potential antigenic targets, they cannot fully predict how a vaccine will behave in vivo. Successful DNA vaccination depends on plasmid delivery into host cells and subsequent antigen expression by the host. These factors can vary depending on the delivery method and the host’s biological characteristics. Differences in feline MHC alleles may also influence epitope presentation and immune responsiveness across populations. Although multiple-sequence alignment and comparison of 112 isolates strengthen epitope homology, the genetic diversity of circulating FIV strains may still affect the extent to which predicted epitopes remain conserved and capable of eliciting protective immunity. Furthermore, producing a stable vaccine and efficiently delivering it into a feline system presents additional challenges and requires further optimization.

Future studies should experimentally validate the proposed vaccine construct, beginning with in vitro experiments to evaluate protein stability, safety, and initial efficacy. Further in vivo studies can provide a stronger evaluation of safety, immune response, and vaccine effectiveness in a complex system. The vaccine’s potential would then be defined by assessing dosing strategies, particularly against various FIV strain challenges. Immunoinformatics can be used to rapidly update and refine current vaccines as new isolates emerge, particularly in HIV, by streamlining epitope identification and testing multiple properties.

## 5. Conclusions

In conclusion, this study demonstrates that an immunoinformatics-driven approach to vaccine design can produce a stable and effective vaccine targeting FIV in silico. The favorable structural characteristics and strong immune response may indicate this construct’s ability to produce effective immunity in vivo. As for rapidly mutating retroviruses like HIV, this approach shows rapid in silico methods of targeting multiple regions of consistently evolving viruses and may provide an effective platform for the design of future treatments.

## Figures and Tables

**Table 1:** Bioinformatic tools and web servers utilized for predictions.

Software	Version	Release Year	URL	Reference
Geneious	v2023.1	2023	https://www.geneious.com/ (accessed on 5 October 2025)	-
ABCpred	v1.0	2006	https://webs.iiitd.edu.in/raghava/abcpred (accessed on 5 October 2025)	[42]
IEDB	v2.24	2023	https://tools.iedb.org (accessed on 12 October 2025)	[43]
NCBI Blastp	v2.14.1	2023	https://blast.ncbi.nlm.nih.gov/ (accessed on 5 October 2025)	-
Vaxijen	v2.0	2007	https://www.ddg-pharmfac.net/vaxijen/VaxiJen/VaxiJen.html (accessed on 15 October 2025)	[44]
Allertop	v2.1	2014	https://www.ddg-pharmfac.net/AllerTOP (accessed on 10 October 2025)	[45]
Toxinpred	V1.0	2013	https://webs.iiitd.edu.in/raghava/toxinpred (accessed on 10 October 2025)	[46]
TMHMM-2.0	V2.0	2001	https://services.healthtech.dtu.dk/services/TMHMM-2.0/ (accessed on 10 October 2025)	[47]
NETPHOS-3.1	V3.1	2012	https://services.healthtech.dtu.dk/services/NetPhos-3.1/ (accessed on 15 October 2025)	[48,49]
Expasy	-	2024	https://www.expasy.org (accessed on 15 October 2025)	[50]
NetNGlyc 1.0	V1.0	2004	https://services.healthtech.dtu.dk/services/NetNGlyc-1.0/ (accessed on 15 October 2025)	[51]
Alphafold	V2.3	2022	https://alphafold.ebi.ac.uk (accessed on 25 October 2025)	[52,53]
Colabfold	V1.5	2022	https://github.com/sokrypton/ColabFold (accessed on 20 October 2025)	[54]
ProSA	-	2017	https://prosa.services.came.sbg.ac.at (accessed on 15 October 2025)	[55,56]
MolProbity	V4.5	2021	http://molprobity.biochem.duke.edu (accessed on 15 October 2025)	[57]
GalaxyRefine	-	2020	http://galaxy.seoklab.org/refine (accessed on 30 October 2025)	[58,59]
HDock	V1.1	2020	http://hdock.phys.hust.edu.cn (accessed on 30 October 2025)	[60]
PDBePISA	V1.52	2011	https://www.ebi.ac.uk/pdbe/pisa (accessed on 25 October 2025)	[61]
C-Immsim	V1.0	2019	https://150.146.2.1/C-IMMSIM (accessed on 30 October 2025)	[62]
Addgene	-	2024	https://www.addgene.org (accessed on 25 October 2025)	-
Snapgene	V7.2	2024	https://www.snapgene.com (accessed on 5 October 2025)	-
IL4Pred	V1.0	2012	https://webs.iiitd.edu.in/raghava/il4pred (accessed on 15 October 2025)	[63]
IFNEpitope	V1.0	2013	https://webs.iiitd.edu.in/raghava/ifnepitope (accessed on 15 October 2025)	[64]

**Table 2:** Mapping of the B-Cell Epitopes across the key proteins of the FIV genome.

Gene	Epitope (aa)	Antigenicity	Allergenicity	Toxicity	Surface Exposed	Cross- Reactivity
*Gag*	^161^IQTVNGAPQYVALDPK^176^	1.266	Non- allergen	Non-toxin	Yes	No
*Gag*	^332^VKLYLKQSLSIANANP^347^	0.847	Non- allergen	Non-toxin	Yes	No
*Pol*	^744^GEGILDKRAEDAGYDL^759^	1.15	Non- allergen	Non-toxin	Yes	No
*Pol*	^513^GPHQICYQVYQKEGNP^528^	0.742	Non- allergen	Non-toxin	Yes	No
*Env*	^298^KVNISLCLTGGKMLYN^313^	0.873	Non- allergen	Non-toxin	Yes	No
*Env*	^523^KAVEMYNIAGNWSCTS^538^	0.813	Non- allergen	Non-toxin	Yes	No

**Table 3:** Results of the prediction of the top-ranked CTL epitopes across the key FIV proteins.

Gene	Epitope (aa)	Restricting MHC Allele	Antigenicity	Allergenicity	Toxicity	Cross- Reactivity
*Gag*	^55^DLQERREKF^63^	DLA-8803401	1.229	Non- allergen	Non-toxin	No
*Gag*	^138^RMANVSTGR^146^	DLA-8803401	1.108	Non- allergen	Non-toxin	No
*Pol*	^267^SLAVHSLNF^275^	DLA-8803401	1.053	Non- allergen	Non-toxin	No
*Pol*	^397^RMLIDFREL^405^	DLA-8803401	0.967	Non- allergen	Non-toxin	No
*Env*	^379^RTQSQPGSW^387^	DLA-8803401	1.365	Non- allergen	Non-toxin	No
*Env*	^512^YTAFAMQEL^520^	DLA-8803401	1.085	Non- allergen	Non-toxin	No

**Table 4:** Results of the prediction of the top-ranked HTL epitopes across the key FIV proteins.

Gene	Epitope (aa)	Restricting MHC Allele	Antigenicity	Allergenicity	Toxicity	IL-4	IFN-γ	Cross- Reactivity
*Gag*	^134^RWAIRMANVTTGREP^148^	HLA-DRB1*01:01	1.082	Non- allergen	Non-toxin	Yes	No	No
*Gag*	^269^LTQEQQAEPRFAPAR^283^	HLA-DRB1*04:01	0.656	Non- allergen	Non-toxin	Yes	No	No
*Gag*	^217^QLWFTAFSANLTPTD^231^	HLA-DRB1*01:01	0.843	Non- allergen	Non-toxin	Yes	Yes	No
*Pol*	^312^FTQNQQWIGPEEAEE^326^	HLA-DRB1*07:01	0.613	Non- allergen	Non-toxin	Yes	No	No
*Pol*	^401^DPDYAPYTAFTLPRK^415^	HLA-DRB1*11:01	0.933	Non- allergen	Non-toxin	Yes	No	No
*Env*	^386^GSWFRAISSWKQRNR^400^	HLA-DRB1*15:01	0.459	Non-allergen	Non-toxin	Yes	Yes	No

## Data Availability

The original data presented in the study are openly available at the online repository https://doi.org/10.6084/m9.figshare.31301710 (accessed on 5 October 2025).

## Supplementary Materials

The following supporting information can be downloaded at: https://www.mdpi.com/article/10.3390/pathogens15030341/s1, Table S1: Full amino acid sequences of 112 FIV isolates obtained from the NCBI database. Used to generate consensus sequences; Table S2: All valid B, CD4, and CD8 epitopes, respectively; Table S3: Pairwise sequence identity between feline leukocyte antigen (FLA) class I alleles and canine DLA-88 alleles used for MHC I epitope prediction; Table S4: Pairwise sequence identity between feline leukocyte antigen (FLA-DRB) class II alleles and human HLA-DRB alleles used for MHC II epitope prediction; Table S5: Expasy PROTPARAM results; Figure S1: PDBePISA analysis of ligand-TLR9 docking; Figure S2: HDock models and docking scores.
