# Supplementary material for: Multi-Epitope DNA-Based Feline Immunodeficiency Virus Vaccine Construct Designed by Immunoinformatic and Machine Learning Tools as a Surrogate Model for HIV Vaccine Development
Source: Pathogens. 2026 Mar 23;15(3):341. doi: 10.3390/pathogens15030341 (PMC13029239; doi:10.3390/pathogens15030341)
Supplement: Supplementary file 1 [file pathogens-15-00341-s001.zip › pathogens-4171825-supplementary.pdf]

Supplementary Materials

Table S1: Full amino acid sequences of 112 FIV isolates obtained from the NCBI database. Used to generate consensus sequences.

Name	Accession	COO	Date Added
Feline immunodeficiency virus isolate 73, partial genome	OR863713.1	Brazil: Mossoro	24-Jun-2021
Feline immunodeficiency virus isolate 114, partial genome	OR863721.1	Brazil: Mossoro	09-Sep-2021
Feline immunodeficiency virus isolate 81, partial genome	OR863716.1	Brazil: Mossoro	21-Jul-2021
Feline immunodeficiency virus isolate GF6 from Colombia, partial genome	MN630242.1	Colombia: Bogota	2018
Feline immunodeficiency virus isolate 10, complete genome	MW815633.1	Brazil: Botucatu	2008
Feline immunodeficiency virus isolate 9, partial genome	MW142027.1	Brazil: Botucatu	2007
Feline immunodeficiency virus isolate 21, partial genome	MW142037.1	Brazil: Sao Paulo	2011
Feline immunodeficiency virus isolate 27, partial genome	MW142041.1	Brazil: Sao Paulo	2011
Feline immunodeficiency virus isolate 28, partial genome	MW142042.1	Brazil: Sao Paulo	2011
Feline immunodeficiency virus isolate 26, complete genome	MW815635.1	Brazil: Sao Paulo	2011
Feline immunodeficiency virus isolate 12, partial genome	MW142029.1	Brazil: Botucatu	2007
Feline immunodeficiency virus isolate 19, partial genome	MW142035.1	Brazil: Botucatu	2011
Feline immunodeficiency virus isolate 14, partial genome	MW142031.1	Brazil: Botucatu	2007
Feline immunodeficiency virus isolate 20, partial genome	MW142036.1	Brazil: Botucatu	2011
Feline immunodeficiency virus isolate 22, partial genome	MW142038.1	Brazil: Sao Paulo	2011
Feline immunodeficiency virus isolate 24, partial genome	MW142039.1	Brazil: Sao Paulo	2011
Feline immunodeficiency virus isolate 25, partial genome	MW142040.1	Brazil: Sao Paulo	2011
Feline immunodeficiency virus isolate 29, partial genome	MW142043.1	Brazil: Sao Paulo	2011
Feline immunodeficiency virus isolate 30, complete genome	MW815636.1	Brazil: Santos	2012
Feline immunodeficiency virus isolate 1D, partial genome	MW142019.1	Brazil: Suzano	2015
Feline immunodeficiency virus isolate 2B, partial genome	MW142020.1	Brazil: Suzano	2012

Feline immunodeficiency virus isolate 3A, partial genome	MW142021.1	Brazil: Suzano	2007
Feline immunodeficiency virus isolate 3B, partial genome	MW142022.1	Brazil: Suzano	2011
Feline immunodeficiency virus isolate 4A, partial genome	MW142023.1	Brazil: Suzano	2007
Feline immunodeficiency virus isolate 4B, partial genome	MW142024.1	Brazil: Suzano	2009
Feline immunodeficiency virus isolate 5, partial genome	MW142025.1	Brazil: Suzano	2011
Feline immunodeficiency virus isolate 6, partial genome	MW142026.1	Brazil: Suzano	2011
Feline immunodeficiency virus isolate 78, partial genome	OR863715.1	Brazil: Mossoro	20-Jul-2021
Feline immunodeficiency virus isolate 105, partial genome	OR863720.1	Brazil: Mossoro	12-Aug-2021
Feline immunodeficiency virus isolate 18, partial genome	MW142034.1	Brazil: Botucatu	2011
Feline immunodeficiency virus isolate 16, partial genome	MW142032.1	Brazil: Botucatu	2007
Feline immunodeficiency virus isolate 17, partial genome	MW142033.1	Brazil: Botucatu	2007
Feline immunodeficiency virus isolate 34, partial genome	MW142048.1	Brazil: Botucatu	2002
Feline immunodeficiency virus isolate 46, partial genome	OR863710.1	Brazil: Mossoro	18-May-2021
Feline immunodeficiency virus isolate 101, partial genome	OR863718.1	Brazil: Mossoro	09-Aug-2021
Feline immunodeficiency virus isolate 104, partial genome	OR863719.1	Brazil: Mossoro	11-Aug-2021
Feline immunodeficiency virus isolate 33A, partial genome	MW142046.1	Brazil: Sobral	2017
Feline immunodeficiency virus isolate 33B, partial genome	MW142047.1	Brazil: Sobral	2017
Feline immunodeficiency virus isolate 54, partial genome	OR863711.1	Brazil: Mossoro	26-May-2021
Feline immunodeficiency virus isolate 76, partial genome	OR863714.1	Brazil: Mossoro	01-Jul-2021
Feline immunodeficiency virus isolate 94, partial genome	OR863717.1	Brazil: Mossoro	04-Aug-2021
Feline immunodeficiency virus isolate 11, partial genome	MW142028.1	Brazil: Botucatu	2007
Feline immunodeficiency virus isolate 13, partial genome	MW142030.1	Brazil: Botucatu	2007
Feline immunodeficiency virus isolate 15, complete genome	MW815634.1	Brazil: Botucatu	2006
Feline immunodeficiency virus isolate 41, partial genome	OR863709.1	Brazil: Mossoro	18-May-2021
Feline immunodeficiency virus isolate 120, partial genome	OR863722.1	Brazil: Mossoro	15-Sep-2021

Feline immunodeficiency virus isolate 32A, partial genome	MW142044.1	Brazil: Joinville	2014
Feline immunodeficiency virus isolate 32B, partial genome	MW142045.1	Brazil: Joinville	2014
Feline immunodeficiency virus isolate 55, partial genome	OR863712.1	Brazil: Mossoro	27-May-2021
Feline immunodeficiency virus isolate 27B, complete genome	EU117991.1	USA	N/A
Feline immunodeficiency virus isolate 1027, complete genome	EU117992.1	Botswana	N/A
Feline immunodeficiency virus subtype C, complete genome	AF474246.1	Canada: British Columbia	N/A
Feline immunodeficiency virus, PPR isolate, complete genome; (San Diego isolate)	M36968.1	San Diego	N/A
Feline immunodeficiency virus isolate FIV-C36, complete genome	AY600517.1	N/A	N/A
Feline immunodeficiency virus isolate CHN17, complete genome	MF352016.1	China	N/A
Feline immunodeficiency virus isolate SR631, complete genome	EF455613.1	N/A	N/A
Feline immunodeficiency virus isolate SR631B, complete genome	EF455614.1	N/A	N/A
Feline immunodeficiency virus isolate Mc350, complete genome	EF455604.1	N/A	N/A
Feline immunodeficiency virus isolate Gc34, complete genome	EF455603.1	N/A	N/A
Feline immunodeficiency virus isolate YM29, complete genome	EF455607.1	N/A	N/A
Feline immunodeficiency virus isolate YF16, complete genome	EF455608.1	N/A	N/A
Feline immunodeficiency virus isolate Mc121, complete genome	EF455606.1	N/A	N/A
Feline immunodeficiency virus isolate CoLV, complete genome	EF455615.1	N/A	N/A
Feline immunodeficiency virus clone pOma3, complete genome	AY713445.1	N/A	N/A
Feline immunodeficiency virus isolate JM01, complete genome	EF455609.1	N/A	N/A
Feline immunodeficiency virus isolate Mc100, complete genome	EF455605.1	N/A	N/A
Feline immunodeficiency virus isolate YF125, complete genome	EF455612.1	N/A	N/A
Feline immunodeficiency virus isolate JF6, complete genome	EF455610.1	N/A	N/A
Feline immunodeficiency virus isolate YM137, complete genome	EF455611.1	N/A	N/A

Feline immunodeficiency virus isolate PLV-1695, complete genome	DQ192583.1	Canada: Vancouver Island	N/A
Feline immunodeficiency virus isolate cat/Brazil/Pequeno_2013, partial genome	MF370550.1	Brazil: Suzano	2013
Feline immunodeficiency virus vif gene for viral infectivity factor, complete cds, strain: Shizuoka	LC079040.1	Japan	N/A
Feline immunodeficiency virus USIL2489_7B gag polyprotein (gag) gene, complete cds, polymerase polyprotein (pol) gene, partial cds, vif protein (vif), complete cds, and envelope glycoprotein (env), complete cds, complete genome	U11820.1	N/A	N/A
Feline immunodeficiency virus genomic RNA for GAG protein, POL protein, ENV protein and 3 ORF's	X57002.1	Zurich, Switzerland	N/A
Feline immunodeficiency virus strain FIV-Oma proviral DNA, complete sequence	U56928.1	N/A	N/A
Feline immunodeficiency virus gag polyprotein, complete CDS; pol polyprotein, 3' end; vif protein, complete CDS; and env polyprotein, complete CDS	M59418.1	N/A	N/A
Composition and method for protecting animal from lentivirus-associated diseases and novel feline immunodeficiency virus	E43300.1	N/A	N/A
Compositions and Methods for Protecting Animals from Lentivirus-Associated Disease Such as Feline Immunodeficiency Virus	DD029017.1	N/A	N/A
X	DL231479.1<br>X	N/A	N/A
Feline immunodeficiency virus isolate Lru7 gag protein (gag) gene, complete cds; pol protein (pol) gene, partial cds; and vif protein (vif) and env protein (env) genes, complete cds	KF906149.1	USA: Southern California	2005
Feline immunodeficiency virus isolate Lru10 gag protein (gag) gene, complete cds; pol protein (pol) gene, partial cds; and vif protein (vif) and env protein (env) genes, complete cds	KF906152.1	USA: Southern California	2006
Feline immunodeficiency virus isolate Lru1 gag protein (gag) gene, complete cds; pol protein (pol) gene, partial cds; and vif protein (vif) and env protein (env) genes, complete cds	KF906143.1	USA: Southern California	1996
Feline immunodeficiency virus isolate Lru11 gag protein (gag) gene, complete cds; pol protein (pol) gene, partial cds; and	KF906146.1	USA: Southern California	2002

vif protein (vif) and env protein (env) genes, complete cds			
Feline immunodeficiency virus isolate Pco17 gag protein (gag) gene, complete cds; pol protein (pol) gene, partial cds; and vif protein (vif), orfA protein (orfA), and env protein (env) genes, complete cds	KF906146.1	USA: Southern California	2003
Feline immunodeficiency virus isolate Pco2 gag protein (gag) gene, complete cds; pol protein (pol) gene, partial cds; and vif protein (vif), orfA protein (orfA), and env protein (env) genes, complete cds	KF906185.1	USA: Southern California	2002
Feline immunodeficiency virus isolate Pco6 gag protein (gag) gene, complete cds; pol protein (pol) gene, partial cds; and vif protein (vif) and env protein (env) genes, complete cds	KF906168.1	USA: Southern California	2003
Feline immunodeficiency virus isolate Pco7 gag protein (gag) gene, complete cds; pol protein (pol) gene, partial cds; and vif protein (vif) and env protein (env) genes, complete cds	KF906169.1	USA: Southern California	2007
Feline immunodeficiency virus isolate Lru8 gag protein (gag) gene, complete cds; pol protein (pol) gene, partial cds; and vif protein (vif) and env protein (env) genes, complete cds	KF906150.1	USA: Southern California	2006
Feline immunodeficiency virus isolate Lru12 gag protein (gag) gene, complete cds; pol protein (pol) gene, partial cds; and vif protein (vif) and env protein (env) genes, complete cds	KF906154.1	USA: Southern California	2009
Feline immunodeficiency virus isolate Lru9 gag protein (gag) gene, complete cds; pol protein (pol) gene, partial cds; and vif protein (vif) and env protein (env) genes, complete cds	KF906151.1	USA: Southern California	2007
Feline immunodeficiency virus isolate Lru19 gag protein (gag) gene, complete cds; pol protein (pol) gene, partial cds; and vif protein (vif) and env protein (env) genes, complete cds	KF906161.1	USA: Florida	2010
Feline immunodeficiency virus isolate Pco5 gag protein (gag) gene, complete cds; pol protein (pol) gene, partial cds; and vif protein (vif) and env protein (env) genes, complete cds	KF906167.1	USA: Southern California	2004

Feline immunodeficiency virus isolate Lru6 gag protein (gag) gene, complete cds; pol protein (pol) gene, partial cds; and vif protein (vif) and env protein (env) genes, complete cds	KF906148.1	USA: Southern California	2003
Feline immunodeficiency virus isolate Lru11 gag protein (gag) gene, complete cds; pol protein (pol) gene, partial cds; and vif protein (vif) and env protein (env) genes, complete cds	KF906153.1	USA: Southern California	2009
Feline immunodeficiency virus isolate Lru5 gag protein (gag) gene, complete cds; pol protein (pol) gene, partial cds; and vif protein (vif) and env protein (env) genes, complete cds	KF906147.1	USA: Southern California	2002
Feline immunodeficiency virus isolate Pco8 gag protein (gag) gene, complete cds; pol protein (pol) gene, partial cds; and vif protein (vif) and env protein (env) genes, complete cds	KF906170.1	USA: Southern California	2002
Feline immunodeficiency virus isolate Lru15 gag protein (gag) gene, complete cds; pol protein (pol) gene, partial cds; and vif protein (vif) and env protein (env) genes, complete cds	KF906157.1	USA: Florida	2007
Feline immunodeficiency virus isolate Lru2 gag protein (gag) gene, complete cds; pol protein (pol) gene, partial cds; and vif protein (vif) and env protein (env) genes, complete cds	KF906144.1	USA: Southern California	2001
Feline immunodeficiency virus isolate Lru16 gag protein (gag) gene, complete cds; pol protein (pol) gene, partial cds; and vif protein (vif) and env protein (env) genes, complete cds	KF906158.1	USA: Florida	2010
Feline immunodeficiency virus isolate Lru17 gag protein (gag) gene, complete cds; pol protein (pol) gene, partial cds; and vif protein (vif) and env protein (env) genes, complete cds	KF906159.1	USA: Florida	2010
Feline immunodeficiency virus isolate Lru3 gag protein (gag) gene, complete cds; pol protein (pol) gene, partial cds; and vif protein (vif) and env protein (env) genes, complete cds	KF906145.1	USA: Southern California	2001
Feline immunodeficiency virus isolate Lru14 gag protein (gag) gene, complete cds; pol protein (pol) gene, partial cds; and vif protein (vif) and env protein (env) genes, complete cds	KF906156.1	USA: Florida	1984

Feline immunodeficiency virus isolate Pco1 gag protein (gag) gene, complete cds; pol protein (pol) gene, partial cds; and vif protein (vif) and env protein (env) genes, complete cds	KF906163.1	USA: Southern California	2004
Feline immunodeficiency virus isolate Lru20 gag protein (gag) gene, complete cds; pol protein (pol) gene, partial cds; and vif protein (vif) and env protein (env) genes, complete cds	KF906162.1	USA: Florida	2010
Feline immunodeficiency virus isolate Lru13 gag protein (gag) gene, complete cds; pol protein (pol) gene, partial cds; and vif protein (vif) and env protein (env) genes, complete cds	KF906155.1	USA: Southern California	2009
Feline immunodeficiency virus isolate T90 Gag (gag), Pol (pol), and gp120 (env) genes, partial cds	AH004414.1	N/A	N/A
Feline immunodeficiency virus isolate Pco3 pol protein (pol) gene, partial cds; and vif protein (vif) and env protein (env) genes, complete cds	KF906165.1	USA: Southern California	2009
Feline immunodeficiency virus isolate Lru18 gag protein (gag) gene, complete cds; pol protein (pol) gene, partial cds; and vif protein (vif) and env protein (env) genes, complete cds	KF906160.1	USA: Florida	2010
Feline immunodeficiency virus isolate Lru19 gag protein (gag) gene, complete cds; pol protein (pol) gene, partial cds; and vif protein (vif) and env protein (env) genes, complete cds	KF906161.1	USA: Florida	2010
Feline immunodeficiency virus isolate Lru20 gag protein (gag) gene, complete cds; pol protein (pol) gene, partial cds; and vif protein (vif) and env protein (env) genes, complete cds	KF906162.1	USA: Florida	2010
Feline immunodeficiency virus isolate Lru16 gag protein (gag) gene, complete cds; pol protein (pol) gene, partial cds; and vif protein (vif) and env protein (env) genes, complete cds	KF906158.1	USA: Florida	2010

Table S2: All valid B, CD4, and CD8 epitopes, respectively.

Epitope	Start	ABC Pred	Bepi Pred	Antigenicity	Allergenicity	Toxicity	Matches	Exposure	Cross-Reactivity
Gag									
IQTVNGAP QYVALDPK	161	0.86	Moderate	1.2663 (Probable ANTIGEN).	Probable NON-ALLERGEN	Non-Toxin	43	Surface exposed	No
KGVGLRAP QAIAEAYP	145	0.85	Low–Moderate	0.4734 (Probable ANTIGEN).	Probable NON-ALLERGEN	Non-Toxin	0	Surface Exposed	No
VKLYLKQSL SIANANP	332	0.82	Moderate	0.8467 (Probable ANTIGEN).	Probable NON-ALLERGEN	Non-Toxin	51	Surface Exposed	No
CKRAMSHL KPESTLEE	349	0.81	Low	0.4935 (Probable ANTIGEN).	Probable NON-ALLERGEN	Non-Toxin	0	Surface exposed	No
AEPRFAPA RMQCRAW Y	269	0.81	Low	0.7773 (Probable ANTIGEN).	Probable NON-ALLERGEN	Non-Toxin	52	Surface exposed	No
Pol									
GPHQICYQ VYQKEGNP	513	0.93	Low	0.7422 (Probable ANTIGEN).	Probable NON-ALLERGEN	Non-Toxin	0	Surface exposed	No
GEGILDKR AEDAGYDL	744	0.91	Low	1.1498 (Probable ANTIGEN).	Probable NON-ALLERGEN	Non-Toxin	22	Surface exposed	No
QGEIQMDS ERGEKGFG	853	0.9	Low	0.6824 (Probable ANTIGEN).	Probable NON-ALLERGEN	Non-Toxin	40	Surface exposed	No
Env									
SDLPKGWG YMNCNCTN	538	0.93	Strong	0.5610 (Probable ANTIGEN).	Probable NON-ALLERGEN	Non-Toxin	42	Surface exposed	No
FYEIIMDIEQ NNVQGK	747	0.89	Moderate	0.4134 (Probable ANTIGEN).	Probable NON-ALLERGEN	Non-Toxin	48	Surface exposed	No
KVNISLCLT GGKMLYN	298	0.88	Moderate	0.8729 (Probable ANTIGEN).	Probable NON-ALLERGEN	Non-Toxin	52	Surface exposed	No
YTVIAMPEI DDEEVHL	823	0.84	Low	0.7663 (Probable ANTIGEN).	Probable NON-ALLERGEN	Non-Toxin	20	Surface exposed	No
KAVEMYNI AGNWSCTS	523	0.84	Strong	0.8133 (Probable ANTIGEN).	Probable NON-ALLERGEN	Non-Toxin	46	Surface exposed	No

Epitope	Start	Allele	Binding Score	Percentile	Antigenicity	Allergenicity	Toxicity	Matches	IFN-γ	IL-4	Cross-Reactivity
Gag											
QLWFTAFSAN LTPTD	171	DRB1*01:01	0.7381	1.5	0.8429 (Probable ANTIGEN).	Probable NON-ALLERGEN	Non-Toxin	7	No	No	No
RWAIRMANVS TGREP	36	DRB1*04:01	0.6759	1.6	1.0490 (Probable ANTIGEN).	Probable NON-ALLERGEN	Non-Toxin	0	No	Yes	No
MVSIFMEKAR EGLGG	153	DRB1*01:01	0.6806	1.8	0.7081 (Probable ANTIGEN).	Probable NON-ALLERGEN	Non-Toxin	0	Yes	No	No
Pol											
LIDFRELNKLT EKGA	101	DRB1*11:01	0.9583	0.08	1.1957 (Probable ANTIGEN).	Probable NON-ALLERGEN	Non-toxin	5	No	Yes	No
DPDYAPYTAF TLPRK	148	DRB107:01; DRB115:01	0.8239	0.41	0.9325 (Probable ANTIGEN).	Probable NON-ALLERGEN	Non-toxin	1	No	No	No
DEGYRGEIGVI MINV	653	DRB1*01:01	0.8711	0.57	0.6863 (Probable ANTIGEN).	Probable NON-ALLERGEN	Non-toxin	2	No	Yes	No

SRKSITLMERQ KIAQ	668	DRB1*11:01	0.8179	0.81	0.8516 (Probable ANTIGEN).	Probable NON-ALLERGEN	Non-toxin	1	No	No	No
MEGVLNYMGV KHKFG	843	DRB1*15:01	0.7069	0.89	0.4225 (Probable ANTIGEN).	Probable NON-ALLERGEN	Non-toxin	0	No	Yes	No
Env											
GSWFRAISSW KQRNR	385	DRB104:01, DRB111:01, DRB107:01, DRB101:01	0.9026	0.2	0.4591 (Probable ANTIGEN).	Probable NON-ALLERGEN	Non-Toxin	0	Yes	Yes	No

Peptide	Start	Allele	Binding Score	Percentile	Antigenicity	Allergenicity	Toxicity	Matches	Cross-Reactivity
Gag									
AVMPSAPPM	435	DLA-8803401	0.515685	0.14	0.4206 (Probable ANTIGEN).	Probable NON-ALLERGEN	Non-Toxin	3	No
KMVSIFMEK	152	DLA-8803401	0.292069	0.47	0.6967 (Probable ANTIGEN).	Probable NON-ALLERGEN	Non-Toxin	57	No
RMANVSTGR	40	DLA-8803401	0.228036	0.69	1.1083 (Probable ANTIGEN).	Probable NON-ALLERGEN	Non-Toxin	0	No
DLQERREKF	66	DLA-8803401	0.1002	1.8	1.2294 (Probable ANTIGEN).	Probable NON-ALLERGEN	Non-Toxin	5	No
Pol									
SLAVHSLNF	894	DLA-8803401	0.596059	0.1	1.0525 (Probable ANTIGEN).	Probable NON-ALLERGEN	Non-toxin	2	No
ALKAGSEEM	513	DLA-8803401	0.383537	0.29	0.5610 (Probable ANTIGEN).	Probable NON-ALLERGEN	Non-toxin	31	No
QISDKIPVV	28	DLA-8803401	0.348168	0.34	0.7291 (Probable ANTIGEN)	Probable NON-ALLERGEN	Non-toxin	1	No
RMLIDFREL	99	DLA-8803401	0.344665	0.35	0.9671 (Probable ANTIGEN).	Probable NON-ALLERGEN	Non-toxin	43	No
ALSLAVHSL	892	DLA-8803401	0.331431	0.37	0.4778 (Probable ANTIGEN).	Probable NON-ALLERGEN	Non-toxin	2	No
VSRKSITLM	667	DLA-8803401	0.297091	0.46	0.8785 (Probable ANTIGEN).	Probable NON-ALLERGEN	Non-toxin	1	No
NVSRKSITL	666	DLA-8803401	0.269176	0.54	0.7814 (Probable ANTIGEN).	Probable NON-ALLERGEN	Non-toxin	1	No
YFSAIPQKL	928	DLA-8803401	0.265864	0.56	0.6597 (Probable ANTIGEN).	Probable NON-ALLERGEN	Non-toxin	4	No
Env									
KVDDLIMHF	509	DLA-8803401	0.819938	0.03	0.5880 (Probable ANTIGEN).	Probable NON-ALLERGEN	Non-Toxin	1	No
RTQSQPGS	379	DLA-	0.254942	0.59	1.3651 (Probable ANTIGEN).	Probable	Non-Toxin	39	No

W		8803401				NON-ALLERGEN			
RLVTLEHQV	666	DLA-8803401	0.180888	0.91	0.5905 (Probable ANTIGEN).	Probable NON-ALLERGEN	Non-Toxin	36	No
ILFIGIIIY	160	DLA-8803401	0.159483	1.1	0.7948 (Probable ANTIGEN).	Probable NON-ALLERGEN	Non-Toxin	0	No
YTAFAMQEL	689	DLA-8803401	0.126388	1.4	1.0848 (Probable ANTIGEN).	Probable NON-ALLERGEN	Non-Toxin	57	No
VLSLVHAF	96	DLA-8803401	0.108448	1.6	0.2433 (Probable NON-ANTIGEN).	Probable NON-ALLERGEN	Non-Toxin	0	No

Table S3: Pairwise sequence identity between feline leukocyte antigen (FLA) class I alleles and canine DLA-88 alleles used for MHC I epitope prediction.

FLA Allele (Felis catus)	DLA Allele (Canis lupus familiaris)	Aligned Length (aa)	Percent Identity (%)
FLA-J (EU915358)	DLA-88*03401	239	74.9
FLA-E (KC763048)	DLA-88*03401	239	72.38
FLA-L (LC534240)	DLA-88*03401	105	77.14
FLA-O (LC534237)	DLA-88*03401	105	73.33
FLA-J (EU915358)	DLA-88*50101	79	20.25
FLA-J (EU915358)	DLA-88*50801	79	20.25
FLA-E (KC763048)	DLA-88*50101	79	16.46
FLA-E (KC763048)	DLA-88*50801	79	16.46
FLA-L (LC534240)	DLA-88*50101	42	16.67
FLA-L (LC534240)	DLA-88*50801	42	16.67
FLA-O (LC534237)	DLA-88*50101	42	14.29
FLA-O (LC534237)	DLA-88*50801	42	14.29

Table S4: Pairwise sequence identity between feline leukocyte antigen (FLA-DRB) class II alleles and human HLA-DRB alleles used for MHC II epitope prediction.

Species	MHC Locus	Allele / Protein ID	Domain Analyzed	Identity to Feline DRB (%)	E-value
Felis catus	FLA-DRB	U51527 / AAB65562.1	DRB β1 extracellular domain	100	–
Homo sapiens	HLA-DRB1	NP_002115.2	DRB β1 extracellular domain	~77–78	$\sim 6\times10^{-33}$
Homo sapiens	HLA-DRB1	DRB1*01:01	DRB β1 extracellular domain	~75–78	$<1\times10^{-32}$
Homo sapiens	HLA-DRB1	DRB1*03:01	DRB β1 extracellular domain	~75–77	$<1\times10^{-32}$
Homo sapiens	HLA-DRB1	DRB1*07:01	DRB β1 extracellular domain	~76–78	$<1\times10^{-32}$
Homo sapiens	HLA-DRB1	DRB1*09:01	DRB β1 extracellular domain	~75–77	$<1\times10^{-32}$
Homo sapiens	HLA-DRB1	DRB1*11:01	DRB β1 extracellular domain	~76–78	$<1\times10^{-32}$
Homo sapiens	HLA-DRB1	DRB1*15:01	DRB β1 extracellular domain	~75–77	$<1\times10^{-32}$

Table S5: Expasy PROTPARAM results.

Parameter	Result
Amino acid length	320 aa
Molecular weight	35.40 kDa
Theoretical pI	9.91
Instability index	29.71
Aliphatic index	64.84
GRAVY score	−0.600
otal positively charged residues (Arg + Lys	48
otal negatively charged residues (Asp + Gl	23
Extinction coefficient (280 nm, oxidized)	63,955 M^{-1} cm^{-1}
Extinction coefficient (280 nm, reduced)	63,830 M^{-1} cm^{-1}

stimated half-life (mammalian reticulocytes	4.4 h
Estimated half-life (yeast, in vivo)	>20 h
Estimated half-life (E. coli, in vivo)	>10 h
Number of cysteine residues	3
Transmembrane regions	None detected
Overall stability classification	Stable

Figure S1: PDBePISA analysis of ligand-TLR9 docking.

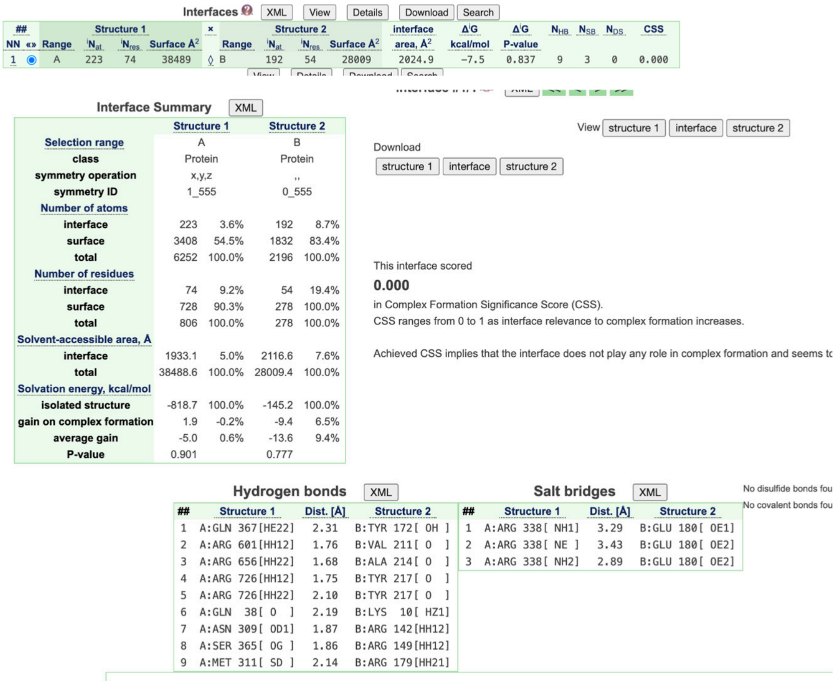

Interfaces XML View Details Download Search

## NN	«»	Structure 1 Range	$^iN_{at}$	$^iN_{res}$	Surface Å²	×	Structure 2 Range	$^iN_{at}$	$^iN_{res}$	Surface Å²	interface area, Å²	Δ^iG kcal/mol	Δ^iG P-value	N_{HB}	N_{SB}	N_{DS}	CSS
1	◉	A	223	74	38489	◊	B	192	54	28009	2024.9	−7.5	0.837	9	3	0	0.000

Interface Summary XML

	Structure 1		Structure 2	
Selection range	A		B	
class	Protein		Protein	
symmetry operation	x,y,z		,,	
symmetry ID	1_555		0_555	
Number of atoms				
interface	223	3.6%	192	8.7%
surface	3408	54.5%	1832	83.4%
total	6252	100.0%	2196	100.0%
Number of residues				
interface	74	9.2%	54	19.4%
surface	728	90.3%	278	100.0%
total	806	100.0%	278	100.0%
Solvent-accessible area, Å				
interface	1933.1	5.0%	2116.6	7.6%
total	38488.6	100.0%	28009.4	100.0%
Solvation energy, kcal/mol				
isolated structure	-818.7	100.0%	-145.2	100.0%
gain on complex formation	1.9	-0.2%	-9.4	6.5%
average gain	-5.0	0.6%	-13.6	9.4%
P-value	0.901		0.777	

View structure 1 interface structure 2

Download structure 1 interface structure 2

This interface scored

0.000

in Complex Formation Significance Score (CSS).

CSS ranges from 0 to 1 as interface relevance to complex formation increases.

Achieved CSS implies that the interface does not play any role in complex formation and seems tc

Hydrogen bonds XML

##	Structure 1	Dist. [Å]	Structure 2
1	A:GLN 367[HE22]	2.31	B:TYR 172[OH]
2	A:ARG 601[HH12]	1.76	B:VAL 211[O]
3	A:ARG 656[HH22]	1.68	B:ALA 214[O]
4	A:ARG 726[HH12]	1.75	B:TYR 217[O]
5	A:ARG 726[HH22]	2.10	B:TYR 217[O]
6	A:GLN 38[O]	2.19	B:LYS 10[HZ1]
7	A:ASN 309[OD1]	1.87	B:ARG 142[HH12]
8	A:SER 365[OG]	1.86	B:ARG 149[HH12]
9	A:MET 311[SD]	2.14	B:ARG 179[HH21]

Salt bridges XML

##	Structure 1	Dist. [Å]	Structure 2
1	A:ARG 338[NH1]	3.29	B:GLU 180[OE1]
2	A:ARG 338[NE]	3.43	B:GLU 180[OE2]
3	A:ARG 338[NH2]	2.89	B:GLU 180[OE2]

No disulfide bonds fou

No covalent bonds fou

Figure S2: HDock models and docking scores.

Summary of the Top 10 Models

Rank	1	2	3	4	5	6	7	8	9	10
Docking Score	-304.77	-296.37	-293.78	-289.30	-287.41	-285.52	-284.11	-284.02	-277.83	-277.75
Confidence Score	0.9567	0.9492	0.9466	0.9419	0.9398	0.9376	0.9360	0.9359	0.9280	0.9279
Ligand rmsd (Å)	47.16	53.63	42.91	58.05	52.77	49.54	64.71	66.44	66.88	69.52
Interface residues	model 1	model 2	model 3	model 4	model 5	model 6	model 7	model 8	model 9	model 10
